# Authentication scheme for routine verification of genetically similar laboratory colonies: a trial with *Anopheles gambiae*

**DOI:** 10.1186/1472-6750-9-91

**Published:** 2009-10-22

**Authors:** Elien E Wilkins, Paula L Marcet, Alice C Sutcliffe, Paul I Howell

**Affiliations:** 1 Entomology, Centers for Disease Control and Prevention (CDC), Atlanta GA, USA; 2 Atlanta Research & Education Foundation (AREF), Veterans Affairs, Atlanta GA, USA

## Abstract

**Background:**

When rearing morphologically indistinguishable laboratory strains concurrently, the threat of unintentional genetic contamination is constant. Avoidance of accidental mixing of strains is difficult due to the use of common equipment, technician error, or the possibility of self relocation by adult mosquitoes ("free fliers"). In many cases, laboratory strains are difficult to distinguish because of morphological and genetic similarity, especially when laboratory colonies are isolates of certain traits from the same parental strain, such as eye color mutants, individuals with certain chromosomal arrangements or high levels of insecticide resistance. Thus, proving genetic integrity could seem incredibly time-consuming or impossible. On the other hand, lacking proof of genetically isolated laboratory strains could question the validity of research results.

**Results:**

We present a method for establishing authentication matrices to routinely distinguish and confirm that laboratory strains have not become physically or genetically mixed through contamination events in the laboratory. We show a specific example with application to *Anopheles gambiae sensu stricto *strains at the Malaria Research and Reference Reagent Resource Center. This authentication matrix is essentially a series of tests yielding a strain-specific combination of results.

**Conclusion:**

These matrix-based methodologies are useful for several mosquito and insect populations but must be specifically tailored and altered for each laboratory based on the potential contaminants available at any given time. The desired resulting authentication plan would utilize the least amount of routine effort possible while ensuring the integrity of the strains.

## Background

Experimental research often relies on the use of genetically similar animals to ensure that results can be generalized and are repeatable. A research animal's value, or its "uniqueness", consists of its known genotype and phenotype. Inadvertent contamination could call into question the validity of research conducted with the same strain over the course of time: genotypic changes can create different phenotypic properties [1,2]. Thus, research results reliant on the genotypic or phenotypic nature of the strain could be invalidated.

Concurrent maintenance of multiple genetically similar laboratory strains requires much attention to detail along with a strict program for guaranteeing that they are reared without contamination. Even with meticulous handling and rearing practices, the possibility of inadvertent contamination still exists [3]. Researchers often consider contamination events only after deviations in research results occur [4,5]. However, by the time laboratory strains are found to be contaminated, research time and effort have already been consumed. Thus, using routinely authenticated strains with a known genetic pedigree is worthwhile.

Anopheline colonies may be highly genetically diverse when initially established, but over time diversity decreases [6,7]. Continual inbreeding of a population inevitably leads to loss of genetic diversity, or genetic deterioration [8]. Therefore, after a number of generations, laboratory strains could be considered unique "monomorphic" entities representing a specific time, place, and phenotype. In the laboratory, establishment of near monomorphic lines, or phenotypic and genotypic homogenization has importance in reproducible research and is also of great benefit when attempting to establish discrimination methods. The colonization process often results in fixed differences in highly conserved genes that can be used as genetic markers: for example, single nucleotide polymorphisms (SNPs) or established chromosomal arrangements emerging from an originally polymorphic wild population.

Some genetic methodologies to differentiate colonized research animal strains earned publication [9-11], yet none for routine discrimination of conspecific mosquito laboratory strains exist. We present a simple methodology for the routine authentication of laboratory strains reared in the same space involving a combination of standardized techniques that, when applied together, provide assurance of the absence of strain contamination. We illustrate with a specific example of 16 standard lines of *Anopheles *mosquitoes housed at the Malaria Research and Reference Reagent Resource Center (MR4, ).

## Results

### Development of authentication matrix

To develop an authentication matrix, a survey of all strains for known, available distinguishing characteristics is done initially. Considering the MR4 holdings, many strains are morphologically distinct from all others maintained such as *An. dirus*, *An. minimus*, or *An. freeborni*. For those, authentication standards were prepared based upon adult morphology. The remaining strains were sorted by species (i.e. *An. gambiae s.s*. - henceforth referred to as *An. gambiae*) and their known (as observed over time in our laboratory) genotypic/phenotypic traits were compiled. As an example, if the *red-stripe *character was noted in one strain, the remaining strains were surveyed for that character.

To create a matrix, the authentication methods initially identified were ordered from least to greatest in terms of cost and/or time consumption (see Table 1). Once the techniques applied generated a unique resulting combination, it was then possible to distinguish a cohort from all others available in the repository and to consider authentication complete for that strain. The objective of any authentication should be confidence to the level of the individual, rather than cohort, when possible. Methods were then analyzed to see where assays could be combined to save time and resources, aiming to complete authentication in the least number of steps (or in the least amount of time/cost) with a high degree of confidence.

**Table 1:** Authentication table example for *An. gambiae*

	**Morphological Assay**			**Insecticide Assay**				**Molecular Assay**		
**Test**	**mel**	**eye**	**c+/cc**	**Perm**	**Diel**	**DDT**	**Prop**	**2La:TEP1**	**GA/rDNA**	***white***

Marker	1	2	3	4	5	6	7	8	9	10
4ARR	no	pink								
M2	no	white								
RMOSP	no	mosaic								
MALI	yes	wild	c+	no	no	no	no	2La/2La:r	GA/M	Mali
RSP-ST	yes	wild	c+	yes	no	no	no	2L+^a^/2L+^a^:s		
AKRON	yes	wild	c+	no	no	no	yes			
SUA	yes	wild	c+	no	no	no	no	2La/2La:s		
PIMPER	yes	wild	c+	no	no	no	no	2L+^a^/2La:r/s		Pimper
MOPTI	yes	wild	c+/cc	no	no	no	no	2La/2La:r	GA/M	Mopti
ZAN/U	yes	wild	c+/cc	no	no	yes				Zan/u
G3	yes	wild	c+/cc	no	no	no	no	2L+^a^/2La:s	GA/MS	
IN22C+	y/n*	wild	c+/cc	no	yes					
RSP	yes	wild	c+/cc	yes						
KISUMU	yes	wild	c+/cc	no	no	no	no	2L+^a^/2La:s	GA/S	
ASEMBO	yes	wild	cc	no	no	no	no	2L+^a^/2L+^a^:s		
L3-5	yes	wild	cc	no	no	no	no	2L+^a^/2L+^a^:r		

Authentication table for MR4 An. gambiae s.s. colonies detailing morphological and molecular characteristics useful for differentiation. Order of tests from left to right: mel: Homochromy (larval cuticular darkening), eye color (pupal or adult), c+/cc (collarless trait), permethrin resistance, dieldrin resistance, DDT resistance, propoxur resistance, PCR: 2La and TEP1, PCR: An. gambiae species identification and rDNA, PCR: white gene. * IN22C+ population is polymorphic for this trait.

**Figure 1:**
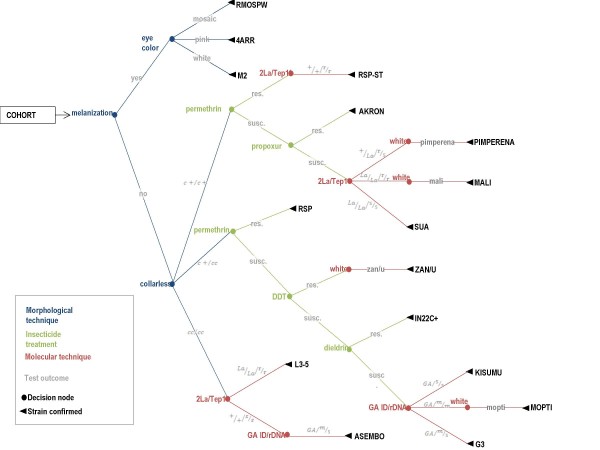
**Flow chart for authentication of *An. gambiae s.s*. strains housed at the MR4**. This chart starts from an unknown cohort of mosquitoes. Insecticide testing is always conducted on a subset. All groups are scanned morphologically for confirmation of *An. gambiae *features. susc. = susceptible, res. = resistant.

Once the matrix is completed, each line of the table (see Table 1) represents an authentication method for a particular strain. Additionally, a flow chart was created that represents how an unknown cohort of *An. gambiae *reared at the MR4 can be distinguished (Figure 1). This decision tree presents a different way of thinking about the same authentication scheme. Of note, authentications must be conducted on a cohort, see discussion.

### Phenotypic discrimination

Phenotypic markers are highly useful in routine strain authentication, when available, because phenotypic examination is quick and inexpensive. Even if these markers are not practical as a unique strain identifier, in combination with other methods, they may limit the total number of steps necessary. We employed only phenotypic traits that had been observed over years in our laboratories and had been previously reported and described by other researchers. Development of new phenotypic markers as identifying characteristics requires extensive knowledge about the inheritance of characters. The traits employed in the MR4 authentication matrix follow.

#### Homochromy (cuticle darkening)

**Figure 2:**
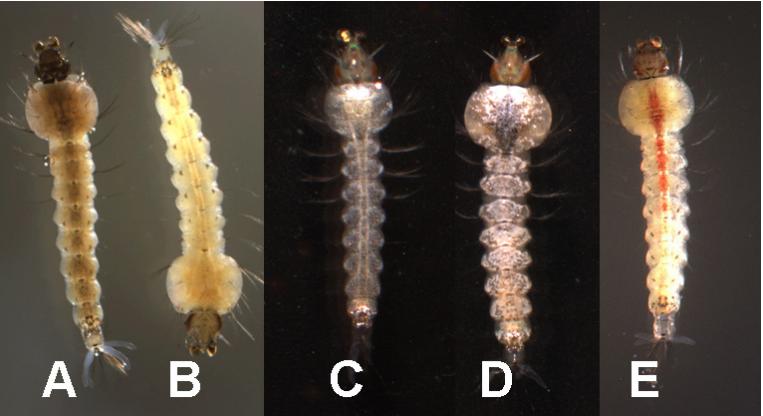
**Phenotypic characteristics in some MR4 *An. gambiae *colonies**. Example of homochromy of wild type larvae when reared in a black-bottom pan (**A**) as opposed to a white-bottom pan (**B**); c+ or collared individual (**C**); black diamond trait (shown just below collar, **D**); red stripe (**E**), only on female larvae.

Non-wild eye strains (i.e. eye color mutants) will not melanize when cultured in a dark-bottomed pan while the opposite is true for wild eye strains [12,13]. The MR4 currently houses several eye color mutants therefore all *An. gambiae *were reared in a dark-bottomed pan parallel to counterparts reared in a white-bottomed pan until the fourth instar. At this point, the larvae from the dark bottomed pan were transferred to a white-bottomed pan and the two groups were compared. Among the stocks kept at MR4, the larval cuticles of 4ARR, M2 and RMOSPW (eye color mutants) reared in dark pans were not different in appearance to those reared in white pans while all the remaining strains display homochromy save one. (Figure 2A &2B). The MR4 maintains IN22C+ which has wild eye individuals which do not melanize necessitating further screenings (c+ individuals are typically resistant to dieldrin and melanize while cc individuals which are susceptible to dieldrin and do not melanize).

#### Eye Color

All *An. gambiae *strains were examined for eye color in the pupal or adult stages because both wild type and mutant strains are reared in this facility. The eye color mutants reared at the MR4 are not genetically dominant; therefore any introgression of foreign DNA would lead to a reversion of the eye color phenotype to the wild-type state. Among the stocks kept at MR4, 4ARR, M2, and RMOSPW have unique eye color mutations compared to a wild-type strain exhibiting pink, white, and mosaic (red and white mixed) colors respectively.

#### Collarless

Following the method of Benedict et al [14], the *collarless *trait was employed as an additional phenotypic marker. The *collar *trait presents as a white spot on the dorsal side of the larval thorax and is easy to observe in a black-bottom pan (Figure 2C). This phenotypic character was used to separate the strains into three groups: **c+**: all larvae display the distinctive white patch (SUA2La, RSP-ST, MALI-NIH, PIMPERENA, and AKRON), **c+/cc**: on the cohort level, some larvae have the white patch and others do not (RSP, ZAN/U, IN22C+, KISUMU, MOPTI, G3), and pure-breeding for **cc**: the white patch is absent from all larvae (L3-5 and ASEMBO1).

#### Insecticide resistance

The use of insecticide resistance assays serves the purpose of authentication as well as guaranteeing resistance thresholds. Over time, resistance levels in a strain can decline therefore routine treatment is necessary. After authentication, only individuals surviving the treatment are used for strain propagation.

### Genotypic discrimination

**Figure 3:**
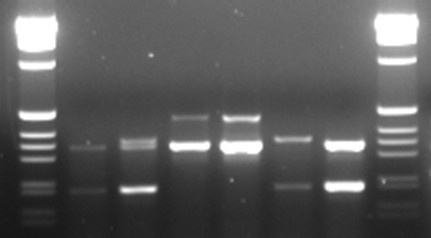
**2La and TEP1 combined PCR assay**. Gel electrophoresis of combined 2La and TEP1 assays. Lanes: (1 and 8) 1 kb ladder marker, (2) TEP1 refractory and 2L+^a ^(L3-5), (3) TEP1 refractory/susceptible and 2L+^a ^(4ARR), (4-5) TEP1 refractory and 2La (MOPTI), (6) TEP1 susceptible and 2L+^a ^(ASEMBO1), (7) TEP1 refractory and 2L+^a ^(L3-5). Not all possible combinations shown.

**Figure 4:**
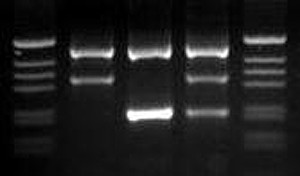
**Combined *An. gambiae *species identification and mopti/savanna rDNA assay**. Gel electrophoresis of An. *gambiae *species identification combined with Mopti - Savanna rDNA assay. Lanes: (1 and 5) 1kb ladder marker, (2) *An. gambiae *MOPTI, (3) *An. gambiae *KISUMU (Savanna), (4) *An. gambiae *ASEMBO (mixed Mopti and Savanna types [38].

**Figure 5:**
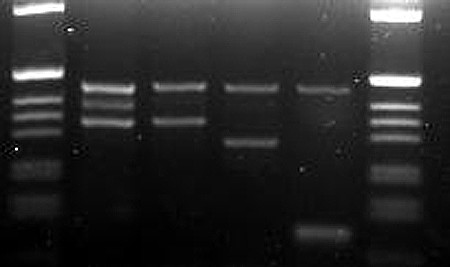
***White *gene SNP PCR assay**. Gel electrophoresis of *An. gambiae white *gene PCR assay. Lanes (1 and 6) 1kb ladder marker, (2) MOPTI, (3) ZAN/U, (4) MALI, (5) PIMPERENA.

Screening for genetic markers is more time consuming and costly than the previously mentioned methods. However, for many of the strains housed at the MR4, the value of the strain depends on fixed genetic characters; so this method of screening is logical and necessary. The first criteria for molecular characteristics in our scenario were those that were of distinct importance to the value of our strains such as the refractory nature of the thioester-containing protein gene for L3-5 or the 2La chromosomal arrangement for SUA2La. It was determined that these two established PCR methods (TEP1 [15] and 2La [16]) could be multiplexed reducing the time, cost, and effort (Figure 3). Similarly, SNPs commonly associated with the Mopti/Savanna (M/S) rDNA types were previously used to create a molecular assay combined with an established method for *An. gambiae *species identification assay as described in Wilkins et al. [17] (Figure 4). The *white *gene was also useful in separating genetically similar MOPTI, MALI and ZAN/U, PIMERPENA using strain-specific SNPs (Figure 5).

### Quantities required for establishment of traits and routine testing

The colonies illustrated in this example have been maintained for many years and the traits tested extensively. For initial screenings of new colonies, it is recommended that 100% of the individuals be examined for each characteristic for no less than 5 sequential generations and continuing in a recurring pattern (every nth generation, based on laboratory constraints) to ensure stability and reproducibility of characters and testing mechanics. For each laboratory and situation, the quantity of individuals to test routinely should also be based on frequency of testing and laboratory constraints. For morphological traits or insecticide resistant strains, we screen 100% of the individuals every fifth generation due to ease of inspection. Screening all individuals provides the most confidence but is not necessarily imperative if testing occurs frequently. For molecular traits, no less than 48 individuals are tested every fifth generation in our laboratories. Required numbers were chosen somewhat arbitrarily by our group based on laboratory constraints, feasibility, and the animal of interest. However, recurring and frequent testing over the course of generations is most important.

### Record keeping

An authentication record of laboratory strains is useful for historical assurance that uniqueness has not been compromised. Conversion of the chosen authentication methodologies into records is important. At the MR4, each line of Table 1 was converted into a stock-specific authentication form including dates, generation number, and specific criteria (how many individuals should be viewed, expected result, etc.). The observer is responsible for signing and dating a record log after the completion of each assay. If any result is incomplete or ambiguous, the process begins anew with the next generation. Once all assays are completed, the paperwork is archived and available for future researchers. A full evaluation of each strain should be conducted in a recurring fashion determined by the individual researcher. Storing individual insect samples after each authentication evaluation may be useful in the future for determining the timeline of any genetic changes. Archived material may also be valuable for future determinations of whether newly discovered alleles have always been present or if, and possibly when, a strain's genetic makeup may have changed, for example.

By employing an assortment of markers, an authentication matrix such as this one (Table 1, Figure 1) can be established to discriminate large or small numbers of strains. At the MR4, similar matrices are kept for all morphologically indistinguishable strains.

## Discussion

Phenotypic characteristics are most commonly employed for strain discrimination due to ease and cost efficiency. Knowledge of how strains normally behave (larval resting postures, growth rate, size, feeding preferences) serves as an ongoing incomplete authentication process. Observation is also an important part of colony health maintenance as observable factors such as slow growth are a quick indicator of unhealthy conditions or potential contamination. For both reasons, regular monitoring of all strains being housed should be performed by trained personnel, and any unexpected behavioral observations should be evaluated with authentication testing. Nitzki et al. [5], for example, showed practical application of phenotypic markers alerting of genotypic introgression between laboratory populations.

Fixed phenotypic characteristics such as insecticide resistance or visible traits can be used to easily, inexpensively, and routinely scan for contamination events if available in the strains. For example, larvae of *An. gambiae *often express visible characteristics such as *collarless*, *red*-*stripe*, and *black diamond *[18] (Figure 2) which may be unique to certain strains. These same characteristics can be selected out of a polymorphic group as a strain-specific marker [19]. It is important to note that deviations in phenotypic expression are not always a result of inter-strain contamination but can be caused by changes in the rearing environment, for example. Also, the expression of some traits can be variable making it difficult to use for routine authentication. For example, the *collarless *trait can be weakly expressed, making it more difficult to confirm. For these reasons, using genetic markers in concert with phenotypic markers is advisable where possible. Detailed information about the phenotypic characteristics of *An. gambiae *at the MR4 is not presented here but was considered in the development of these methods.

Routine challenge of an insecticide resistant strain removes any potential susceptible contaminants and maintains the strain's insecticide resistance levels which is of particular interest to our laboratory's research apart from authentication methods making it a very useful tool since we would be conducting this selection process anyway. Exposing larvae to an insecticide is an advantageous method of treatment as the concentration is more uniform in solution and all individuals remain in constant contact with the insecticide eliminating the threat of individual underexposure. Resistant populations should always be tested concomitantly with susceptible populations as a control, and if susceptible strains are reared alongside resistant populations, their susceptible nature should be monitored as well.

PCR was our chosen method for genetic discrimination because it can be standardized and reproduced in most laboratories, and it was, therefore, favored over more complicated (Luminex) or technical (karyotyping/allozyme) methods evaluated.

In many animal groups, the use of isogenic lines, or clones, are employed to overcome inter-laboratory variation since individuals tested are genetically homogenous. However the development of these lines involves sibling inter-mating resulting in inbreeding depression [2,3]. The deleterious effects on longevity and fecundity are undesirable in routine colony maintenance [20]. Standard lines, including phenotypically unique strains as well as "wild type" strains, are more often chosen due to their ease in maintenance and higher fitness levels. In higher orders, there is some debate of whether research employing standard lines is valid due to variable genotypes [3]. This is also of concern for culicids [21,22]. However, with alternate technologies such as microarray expression analyses, genetic function can be directly linked regardless of knowledge of the individual's genotype [23].

Hii et al. [6] and Dejong et al. [24] reported inter-laboratory genetic variability in sub-populations of anopheline strains. Although these differences may be due to genetic drift and selection, contamination cannot be initially excluded. Since genetic contamination can occur without complete mixing of the chromosomes, an assay only detecting one marker on a single chromosome could miss this type of contamination. Therefore, assays should employ multiple markers on multiple chromosomes where possible for a higher level of confidence in results. In the example presented in this paper, we chose molecular markers located on the X, 2L, and 3L chromosomes.

### Alternate methods

We analyzed Luminex technology as an alternative method for authentication. With this technology, large numbers of SNP sites can be simultaneously detected in a gene(s) [25]. By multiplexing a PCR with several genes of interest, we were able to create a successful Luminex procedure for screening samples for authentication (data not presented). However, the PCR plus Luminex procedure was more time consuming and expensive than the 1 or 2 PCR methods required as presented here. If more cost- or time-efficient, Luminex technology could be a valuable authentication tool, assuming adequate SNP sites were available for discrimination. The different genes of interest would need to be limited as well to avoid many preliminary PCR steps.

Allozyme analyses and karyotyping are successfully used to discriminate between conspecific laboratory strains [6,26,27]. These methods, however, require extensive training in sample preparation and analyses making routine usage difficult, especially if simpler, less expensive methods are available.

We also considered insertion patterns of transposable elements (TEs) (i.e. mobile genetic units capable of replicating and spreading in the host genome) which have recently been applied to study genetic differentiation between *An. gambiae *molecular forms [28-32]. Among TEs, Short INterspersed Elements (SINEs) have been extensively used as phylogenetic and population genetic markers in primate taxa and *An. gambiae *[28,30,32]. Despite the discriminative value for our strains detected in a few loci during preliminary research (data not presented), we did not pursue this approach for a number or reasons. Most loci tested were polymorphic in most strains and therefore only a few presented fixed variants with discriminative value. Moreover, most loci presented a high percentage of PCR failure (6-49%). For routine screenings, this was especially important as the absence of any amplification product for a particular sample could indicate the presence of a particular mutation in the primer binding sequence or simply a PCR failure. Also, using this method, common "stutter" bands and shadow bands made routine scoring quite difficult. In addition, the few strains that could be classified with this approach could also be authenticated using other techniques with greater reliability.

## Conclusion

In many laboratories, multiple, conspecific strains are often reared simultaneously with limited separation. Physical separation of strains and their associated rearing instruments is the simplest method to prevent inter-strain contamination. However, implementing this level of perfect separation is often not feasible due to space limitations or other constraints. Routine genetic-based validation protocols are highly recommended, but few laboratories report routinely employing such methods [2,5].

Ultimately, development of routine methods requires a thorough review of which potential contaminants are available in an individual laboratory and what is known about each strain. Tests can then be organized by ease of implementation, cost efficiency, availability of equipment, transferrable protocols, and most importantly, what is important to the particular group. Combining the tests for strain-specific outcome combinations yields a matrix for assurance of genetic isolation. Authentication implementation should occur at a frequency that fits the laboratory environment. Authentication procedures and endpoints, however, should never be considered fixed. Revision may be necessary based on the discovery of new phenotypes, genetic markers, or other methods that could be used to discriminate between strains as well as introduction of new genetically similar colonies into the laboratory.

## Methods

### Mosquito rearing and manipulation

Mosquitoes were obtained from the MR4 (CDC Atlanta, GA USA, Table 2). Larvae were reared at 27°C using a standard method [33] with larval diet from Aquaricare™ (Koi Floating Blend). Two cohorts of approximately 300 second instar larvae from each strain were reared concurrently in white and black bottomed pans in order to elicit the homochromy response as described by Benedict et al [13].

**Table 2:** MR4 *Anopheles gambiae s.s*. strains

**Strain name**	**Catalog no**.	**Origin**	**Donor**
4ARR	MRA-121	†	Frank Collins
AKRON	MRA-913	Benin	Martin Akogbeto
ASEMBO	MRA-186	Kenya	Francis Atieli
G3	MRA-112	Gambia	William Collins
IN22C+	MRA-115	†	Mark Benedict
KISUMU1	MRA-762	Kenya	Vincent Corbel
L3-5	MRA-114	†	Frank Collins
M2	MRA-105	†	Mark Benedict
MALI NIH	MRA-860	Mali	Nora Besansky
MOPTI	MRA-763	Mali	Greg Lanzaro
PIMPERENA	MRA-861	Mali	Nora Besansky
RMOSPW	MRA**-**111	†	Mark Benedict
RSP	MRA-334	Kenya	John Vulule
RSP-ST	MRA-698	‡	Frank Collins/Hilary Ranson
SUA2La	MRA-765	Liberia	Alessandra della Torre
ZAN/U	MRA-594	Zanzibar	Frank Collins/Hilary Ranson

Strains mentioned are presented with MR4 catalog reference numbers, origin, and donor information. † derived from G3; ‡ derived from RSP.

### Insecticide treatment

Cohorts of 600 fourth instar larvae were treated with insecticides as described in the MR4 Methods in *Anopheles *Research Training Manual (section 4.3.1) [34] with final concentrations and exposure times as follows: Permethrin (1 ppm, 24 h), Propoxur (20 ppm, 1 h), Dieldrin (1 ppm, 1 h), DDT (0.4 ppm, 24 h).

### PCR

Mosquito samples were prepared for PCR by the method of Rafferty et al [35]. PCR products were observed by separation on 0.5× TBE agarose gels run in 0.5× TBE buffer at 12 v/cm and fragment sizes were estimated using a 1 kb ladder marker (Invitrogen ^®^). Thermal cycling for all analyses was performed in a Bio-Rad iCycler^®^. *Go-Taq *DNA polymerase and the manufacturer's (Promega^®^) recommended buffer at 1× concentration was used for all reactions. PCR reactions consisted of 0.75 U of *Go-Taq *polymerase, 1 mM MgCl_2_, all primers at 1 μM, 0.08 mM dNTPs and a 25 μl total volume.

#### *An. gambiae *2La inversion and TEP1 multiplex PCR (Figure 3)

2La primers (23A2 Universal, 27A2 2La, DPCross5 2L+^a^) were as reported by [16] and TEP1 Primers were as reported by [15] (TEP1Rev, TEP16Rev, TEP1For modified by author Blandin (personal communication) as AAA GCT ACG AAT TTG TTG CGT CA). PCR cycling was as reported by [15] consisting of melting at 95°C for 5 m followed by 40 cycles of 95°C for 30 s, 52°C for 30 s, and 72°C for 45 s, followed by one cycle of 72°C for 7 m.

#### *An. gambiae *identification and rDNA discrimination PCR (Figure 4)

Primers used in this assay were described by Wilkins et al. [17] with the addition of two unique primers to detect SNP sites commonly associated with M/S type [36] as listed in Table 3. PCR cycling consisted of melting at 95°C for 5 m followed by 30 cycles of 95°C for 30 s, 58°C for 30 s, and 72°C for 30 s, followed by one cycle of 72°C for 5 m.

**Table 3:** Primer sequences

**Molecular Assay Primers**			
***An. gambiae species ID/rDNA PCR***			**(5' to 3') Fragment size (bp)**

IMP-UN*	GCTGCGAGTTGTAGAGATGCG	F	
QD-3T*	GCATGTCCACCAACGTAAAtC**C**	R	*An. quadriannulatus *637
ME-3T*	CAACCCACTCCCTTGACGaT**G**	R	*An. melas *and *merus *529
GA-3T*	GCTTACTGGTTTGGTCGGCAtG**T**	R	*An. gambiae *464
AR-3T*	GTGTTAAGTGTCCTTCTCCgT**C**	R	*An. arabiensis *388
IMP-M1	TAGCCAGCTCTTGTCCACTAGTtT**T**	R	Mopti 333
IMP-S1	CCAGACCAAGATGGTTCGcT**G**	R	Savanna 221

*An. gambiae white *gene PCR			

UFOR	ATTATCTGATGAAGCTTGGAGTCTTTT	F	
UREV	ATGAAAATAAGGAGCTTCCTGGCAT	R	positive control 478
MOPR	CTGTTGTCTTACAGTAGGGTTAtG**T**	R	*An. gambiae *MOPTI 413
MOP2R	AACGTACGACGTATGATCTAACt**GA**	R	*An. gambiae *MOPTI/ZAN/U 350
MALR	CTCATATTCAAGGATGAACACAAtA**C**	R	*An. gambiae *MALI 292
PIMPR	TCAATGACATGACGTTATAATCTGTCtT**T**	R	*An. gambiae *PIMPERENA 116

Primers indicated with (*) obtained from Wilkins et al [17]. F and R indicate forward and reverse orientation. Lower case indicates site of intentional mismatch. Bold nucleotides show the site of the SNP.

#### *White *gene PCR (Figure 5)

Primers used to discriminate SNP sites for MOPTI, MALI, ZAN/U, and PIMPERENA are listed in Table 3. PCR cycling consisted of melting at 95°C for 5 m followed by 35 cycles of 95°C for 30 s, 56°C for 30 s, and 72°C for 30 s, followed by one cycle of 72°C for 10 m.

### Sequencing of *white *gene

In order to obtain SNP sites to discriminate MOPTI from MALI and ZAN/U from PIMPERENA, the *white *gene from these mosquito strains was amplified using primers WG2 and WG5 and conditions as described by Mukabayire et al [37]. PCR products were sequenced and matched those of Genbank:U29485 with the exceptions of SNP sites shown in Table 4. SNP sites were used to design primers using the intentional mismatch primer (IMP) method [17] as shown in Table 3.

**Table 4:** SNP sites used in *white *gene discrimination PCR

**# SNP site**	**11591**	**11767**	**11825-6**	**11885**
U29485	TTACTATGAC	GATTTCTTGT	GCTTGTTAGA	GACGCTTTAC
Mopti	TTACTATGAC	GATTTCTTGT	G**TC**TGTTAGA	GAC**A**CTTAAC
Mali	TTACTATGAC	GAT**G**TCTTGT	GCTTGTTAGA	GACGCTTTAC
Pimperena	TTAC**A**ATGAC	GATTTCTTGT	GCTTGTTAGA	GACGCTTTAC
Zan/u	TTACTATGAC	GATTTCTTGT	G**TC**TGTTAGA	GACGCTTTAC

Specific SNP sites in some MR4 strains of *An. gambiae white *gene. Numbers of SNP sites are based on Genbank U29485. SNP sites shown in bold/underlined.

## Authors' contributions

EW developed the unique molecular assays presented, participated in the creation of the authentication matrix, produced the Tables, and co-drafted the manuscript. PM tested the SINEs as an authentication method and wrote the portion of the paper covering that topic. AS participated in the sequencing of the *white *gene and the creation of several Figures. PH participated in the creation of the authentication matrix and co-drafted the manuscript. All authors read and approved the final manuscript.
